# Genome-wide identification, evolutionary estimation and functional characterization of two cotton *CKI* gene types

**DOI:** 10.1186/s12870-021-02990-y

**Published:** 2021-05-22

**Authors:** Yanlong Li, Yaoyao Li, Yuanyuan Chen, Maojun Wang, Jing Yang, Xianlong Zhang, Longfu Zhu, Jie Kong, Ling Min

**Affiliations:** 1 National Key Laboratory of Crop Genetic Improvement, Huazhong Agricultural University, Wuhan , 430070 Hubei China; 2 College of Life Sciences, State Key Laboratory for Conservation and Utilization of Subtropical Agro-Bioresources, South China Agricultural University, Guangzhou , 510642 Guangdong China; 3 Institute of Economic Crops, Xinjiang Academy of Agricultural Sciences, Xinjiang, 830091 China

**Keywords:** Casein kinase I, Cotton, Evolutionary history, Gene expression

## Abstract

**Background:**

Casein kinase I (CKI) is a kind of serine/threonine protein kinase highly conserved in plants and animals. Although molecular function of individual member of *CKI* family has been investigated in *Arabidopsis*, little is known about their evolution and functions in *Gossypium*.

**Results:**

In this study, five cotton species were applied to study *CKI* gene family in cotton, twenty-two species were applied to trace the origin and divergence of *CKI* genes. Four important insights were gained: (i) the cotton *CKI* genes were classified into two types based on their structural characteristics; (ii) two types of *CKI* genes expanded with tetraploid event in cotton; (iii) two types of *CKI* genes likely diverged about 1.5 billion years ago when red and green algae diverged; (iv) two types of cotton *CKI* genes which highly expressed in leaves showed stronger response to photoperiod (circadian clock) and light signal, and most two types of *CKI* genes highly expressed in anther showed identical heat inducible expression during anther development in tetraploid cotton (*Gossypium hirsutum*).

**Conclusion:**

This study provides genome-wide insights into the evolutionary history of cotton *CKI* genes and lays a foundation for further investigation of the functional differentiation of two types of *CKI* genes in specific developmental processes and environmental stress conditions.

**Supplementary Information:**

The online version contains supplementary material available at 10.1186/s12870-021-02990-y.

## Background

Casein kinase I (CKI) is a serine/threonine specific protein kinase, highly conserved in plant and animal species [1–3]. Compared to other protein kinases, CKI proteins contain four short conserved peptides: HIPXR, LPWQGLKA, EXSRRDD, and LLGPSLEDLF [4]. Due to the broad distribution of the *CKI* gene family members and their substrates, *CKI* genes have been found to participate in many biological activities. In yeast, *CKI* plays important roles in gene expression regulation, vesicle trafficking, cell morphogenesis, cell cycle, cell colonization, and DNA repair [5–8]. In mammals, *CKI* is involved in cell proliferation, cytokine production, and the transduction and regulation of a variety of signaling pathways related to apoptosis and tumor production and development, such as Wnt and Hedgehog [9–11].

The *CKI* family in some plants has been studied. The *CKI* family has been divided into two types, one type is canonical casein kinase 1, another type is plant-specific casein kinase 1 [12, 13]. In *Arabidopsis*, there are 17 members in *CKI* family, among which 13 members are canonical casein kinase 1, belonging to CKI-like (CKL) cluster, and 4 members are plant-specific CKI, belonging to Mut9p-LIKE KINASEs (MLKs) cluster [12]. In rice, there are 9 canonical casein kinase 1 and 6 plant specific casein kinase 1 [13]. Both two types of *CKI* family have important functions in the regulation of a variety of biological processes related to growth and development, as well as various responses to environmental stimuli [14]. In rice, abscisic acid (ABA) and brassinolide caused up-regulation of *OsCKI1* (canonical casein kinase 1), while *OsCKI1* deficiency caused shorter primary roots and fewer lateral and adventitious roots [2]. In *Arabidopsis*, the *casein kinase I-like 2 AtCKL2* and *AtCKL3*, are necessary for ABA to regulate seed germination, root growth and gene expression [15–17], and overexpression of either *AtCK1.3* or *AtCK1.4* delayed flowering under long-day conditions in *Arabidopsis* [13]. In addition, *MLK3*, a plant-specific casein kinase 1, is critical for maintaining proper flowering time [18]. In cotton, *GhCKI* was speculated to regulate not only tapetal programmed cell death (PCD) and anther dehiscence [19], but also somatic embryogenesis by modulating auxin homeostasis [20]. Since the functions of most *CKI* genes in higher plants are largely unknown, their identification and characterization are particularly necessary for understanding their role and to potentially employ them as genetic resources for improving crop plant defense against biotic and abiotic stresses. In cotton, no genome-wide characterization of the *CKI* gene family has been reported so far. Besides, there was no systematic research to trace the origin and divergence of two types *CKI* genes. Fortunately, the recently published cotton species genomic information is a solid foundation for characterizing *CKI* genes at a genome-wide level. Here we have investigated several fundamental questions regarding the *CKI* gene family evolution: (i) the diversity of gene structure and domain architecture of cotton *CKI* family; (ii) the evolutionary expansion in cotton of *CKI* family; (iii) the origin and divergence speculation of *CKI* family; (iv) the expression profiles of the cotton *CKI* genes under different conditions. In summary, we retraced the evolution of the *CKI* genes to better understand their essential elements and thus be able to exploit this knowledge for plant growth and development.

## Results

### Identification and classification of the Casein Kinase I (*CKI*) in *Gossypium*

**Fig. 1:**
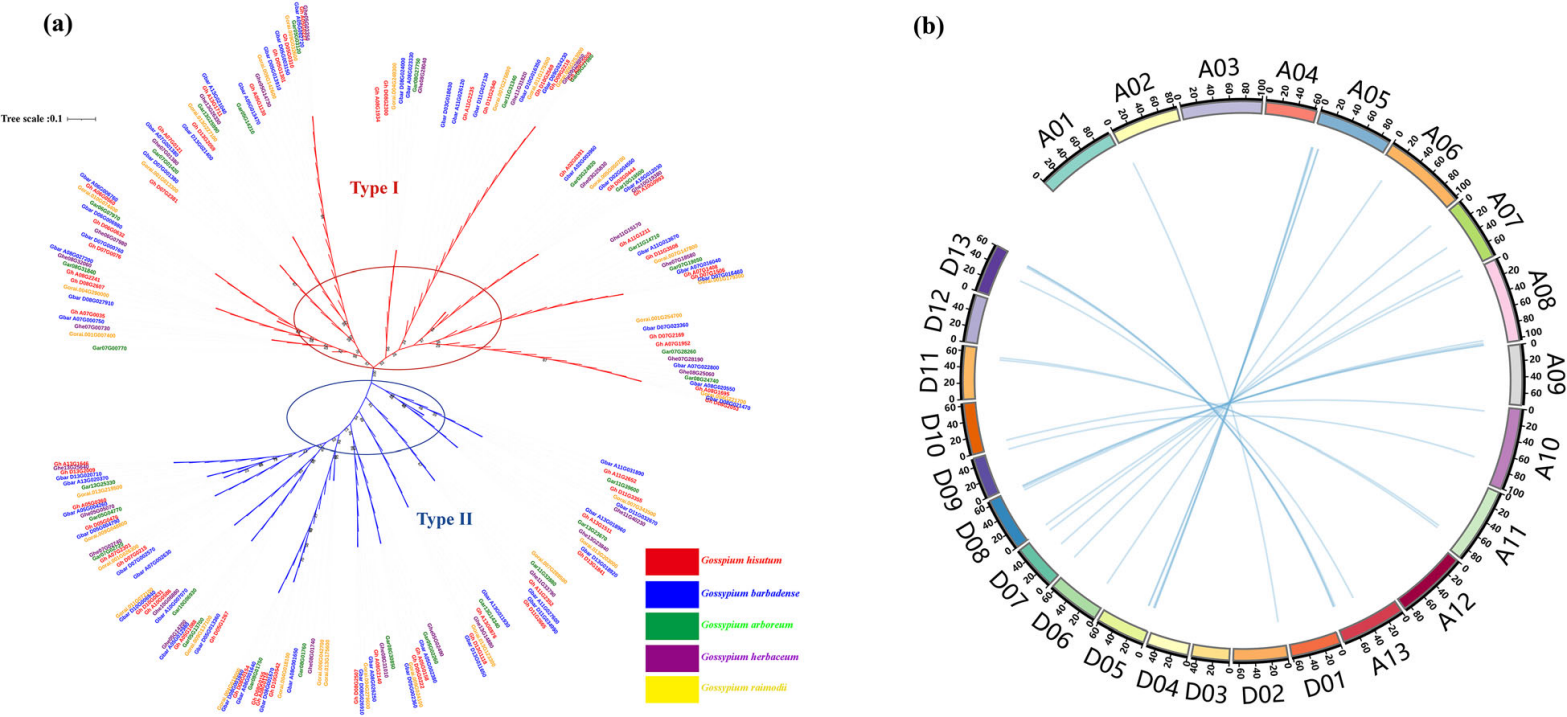
Phylogenetic tree of the Casein Kinase I (*CKI*) in *Gossypium* and chromosomal distribution and gene duplication in *Gossypium hirsutum*. **a** The phylogenetic tree was constructed using 202 cotton CKI protein sequences from *G.hirsutum* (58), *G.arboreum* (30), *G.raimondii* (31), *G.barbadense* (57), *G.herbaceum* (28) with the maximum likelihood (ML) method in MEGA 6. The five different symbols represent the five cotton species: red for *G.hirsutum*, blue for *G.barbadense*, green for *G.arboretum*, purple for *G.herbaceum*, yellow for *G.raimondii*. The Gene ID of *CKI* genes from *G. hirsutum*, *G. arboreum*, *G. raimondii*, *G.barbadense*, *G.herbaceum* were listed in Table S1. **b** The scale is in megabases (Mb), the value on each chromosome represents chromosome length, and the paralogous *GhCKIs* were connected with a blue line

To extract *CKI* sequences in *Gossypium*, the Hidden Markov Model (HMM) was built with 17 *Arabidopsis* CKI protein, then the protein databases of five sequenced cotton species (*G. raimondii*, *G. arboreum*, and *G. hirsutum acc.* TM-1, *G.barbadense*, *G.herbaceum*) [21–24] were extracted with hmmserach. As a result, 31, 29, 27, 58 and 57 CKI members were identified in *G. raimondii* (D genome), *G. arboretum* (A genome), *G.herbaceum* (A genome), *G. hirsutum* (AD genome) and *G.barbadense* (AD genome), respectively (Table S1). To get a better understanding of the phylogenetic relationships between CKIs, an unrooted phylogenetic tree was generated using the CKI protein sequences from *G. raimondii*, *G. arboretum*, *G.herbaceum*, *G. hirsutum* acc. TM-1, *G.barbadense*. Clearly, the CKIs were classified into canonical CKI (named type I) and plant-specific CKI (named type II) in cotton (Fig. 1a). Mostly, a homolog of *CKI* genes can be found once in the diploid *G. raimondii*, once in *G. arboretum,* once in *G.herbaceum*, two copies in the tetraploid *G. hirsutum* acc. TM-1 and two copies in *G.barbadense* 3–79 (Fig. 1a), may indicating the CKI family in tetraploid is derived from A and D diploid.

**Fig. 2:**
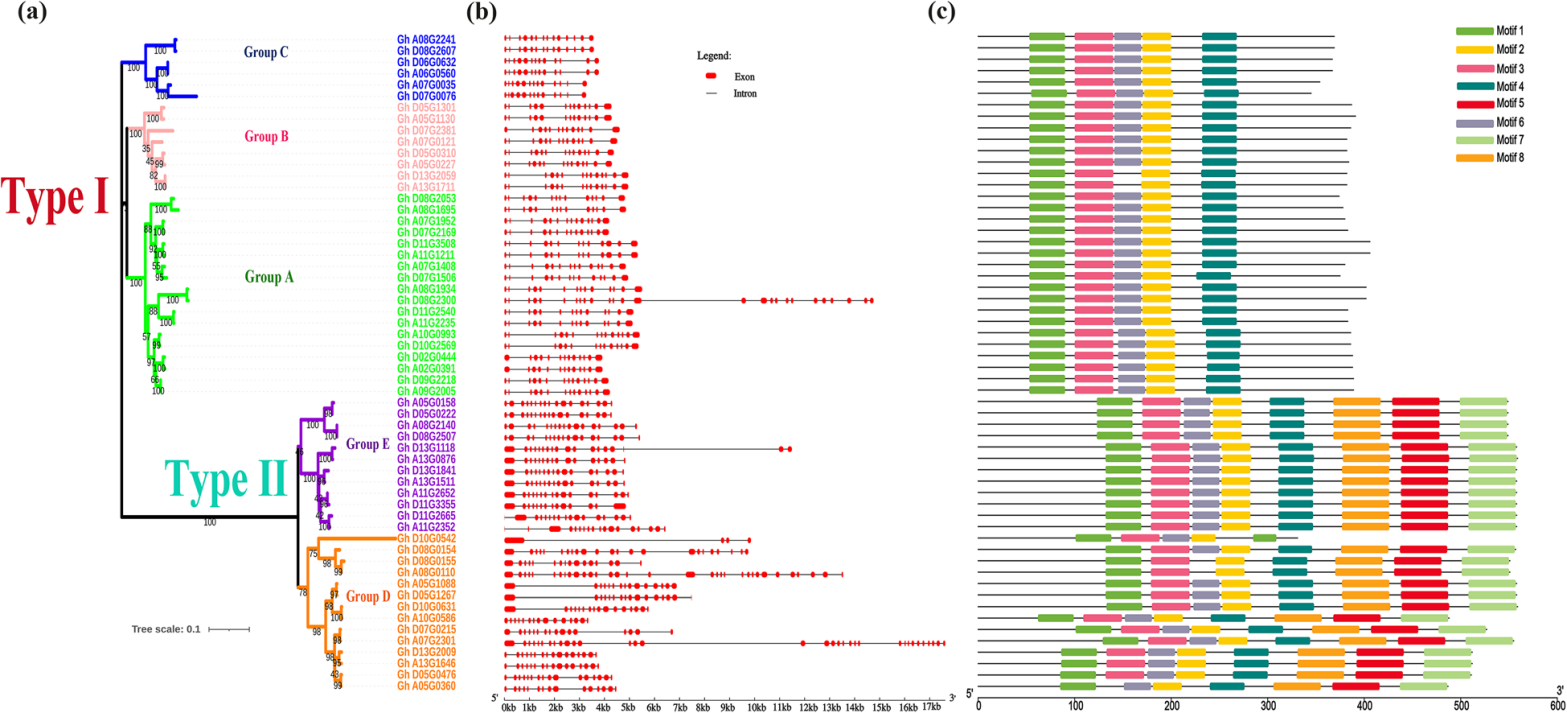
Phylogenetic tree, protein motif and Exon/Intron organization of the Casein Kinase I (*CKI*) in *G. hirsutum.***a** Phylogenetic tree of the Casein Kinase I (*CKI*) in *G.hirsutum* and **b** Exon/Intron organization of the *CKI* genes in *G.hirsutum*. Red boxes represent exons and black lines indicate introns. **c** Motif composition of GhCKI proteins. Conserved motifs in the GhCKI proteins are indicated by colored boxes

Tetraploid cotton species *G. hirsutum*, the most widely cultivated cotton species in the world, are thought to have formed by a polyploidization event that occurred approximately 1–2 million years ago, which involved D and A genome species [25]. Consequently, *G. hirsutum* genome information was used to characterize the location of the *CKI* family genes on chromosome. *GhCKI* were almost evenly distributed on At and Dt subgenomes (Fig. 1b), so the duplication events may illuminate the mechanism about the expansion of *GhCKI* gene family. Then the GhCKI phylogenetic tree was independently constructed using the MEGA 6 software (Fig. 2a). All *G. hirsutum* CKI proteins fell into two distinct groups, which was consistent with the results of Fig. 1a. The type I CKI proteins were further divided into three subclasses: Group A, B, and C; the type II CKI proteins were classified into two subclasses: Group D and Group E.

### Gene structure, conserved motifs and domains of CKIs in *G. hirsutum*

To get a better understanding of the diversification of type I and type II *GhCKI* genes in *G. hirsutum*, the exon/intron organization was analyzed. Most *GhCKI* genes within the same group showed very similar exon/intron distribution patterns in terms of exon length and intron number (Fig. 2b). For example, most type I *GhCKI* genes in groups A, B, and C had thirteen to fifteen exons of similar length, whereas members of type II *GhCKI* genes within group D and E contained more exons. Then, we further analyzed motifs with the program MEME (http://meme-suite.org/tools/meme) to obtain more insights into the diversity of motif compositions among GhCKI proteins. As shown in Fig. 2c, the conserved motifs 1–8 were identified. We clearly observed type I and type II GhCKI proteins have different motif compositions that the type I GhCKI proteins shared five similar motif compositions except Gh_A13G1711 and Gh_D13G2059, but most type II GhCKIs possess eight motifs, implying that GhCKI members within the same type may perform similar functions and that some motifs may play an important role. However, divergence was observed in group E of type II GhCKI, maybe indicating group E members have pluralistic function. Generally, the consistency of motif compositions of GhCKIs with the phylogenetic groups further supported the close evolutionary relationships among GhCKIs, as well as the reliability of our phylogenetic analysis (Fig. 2a). Thus, members belonging to the same group showed similar exon/intron organization and similar motif composition, indicating their functional similarities. And the diverse evolutionary patterns in exon numbers of the *CKI* genes may hint at their functional diversifications in gene expression. These results further supported the classification between type I and type II *GhCKI* genes.

The putative conserved domains of GhCKI proteins in *G. hirsutum* were investigated by using the online program Conserved Domain Search Service (http://www.ncbi.nlm.nih.gov/Structure/cdd/wrpsb.cgi). The type I GhCKI proteins were highly conserved within their kinase domains and N-terminal, but differed significantly in the length and primary structure of C-terminal domains (Figure S1a, Figure S2a). The gene structure of type I GhCKI proteins were consistent with previous reports [3, 4, 26]. The type II GhCKI proteins also presented conserved kinase domains. However, contrary to the type I GhCKI proteins, type II possessed a variable N-terminal and conserved C-terminal (Figure S1b and Figure S2b). In addition, several short sequences are absolutely conserved among the CKIs and not found in other kinases. To further confirm whether the conserved fragments are present in type I and II GhCKI proteins, sequence alignment was performed. The result showed type I GhCKI proteins possess the four short sequences LLGPSLEDLF, HIPXR, EXSRRDD, and LPWQGLKA (Figure S1a). Type II GhCKI proteins contained three of the four conserved sequences (LGPSL, SRRDD, and LPWQG) (Figure S1b). However, compared with the type I CKI proteins, two specific fragments LGKGGFGQV and HGDVKPEN were present in the type II GhCKI proteins only. Comprehensively, LGPSL, SRRDD, and LPWQG appeared in both type I and type II GhCKI proteins. Same result was got in other *Gossypium (G. raimondii*) (Figure S3).

### *CKI* family expanded with tetraploid event in cotton

**Fig. 3:**
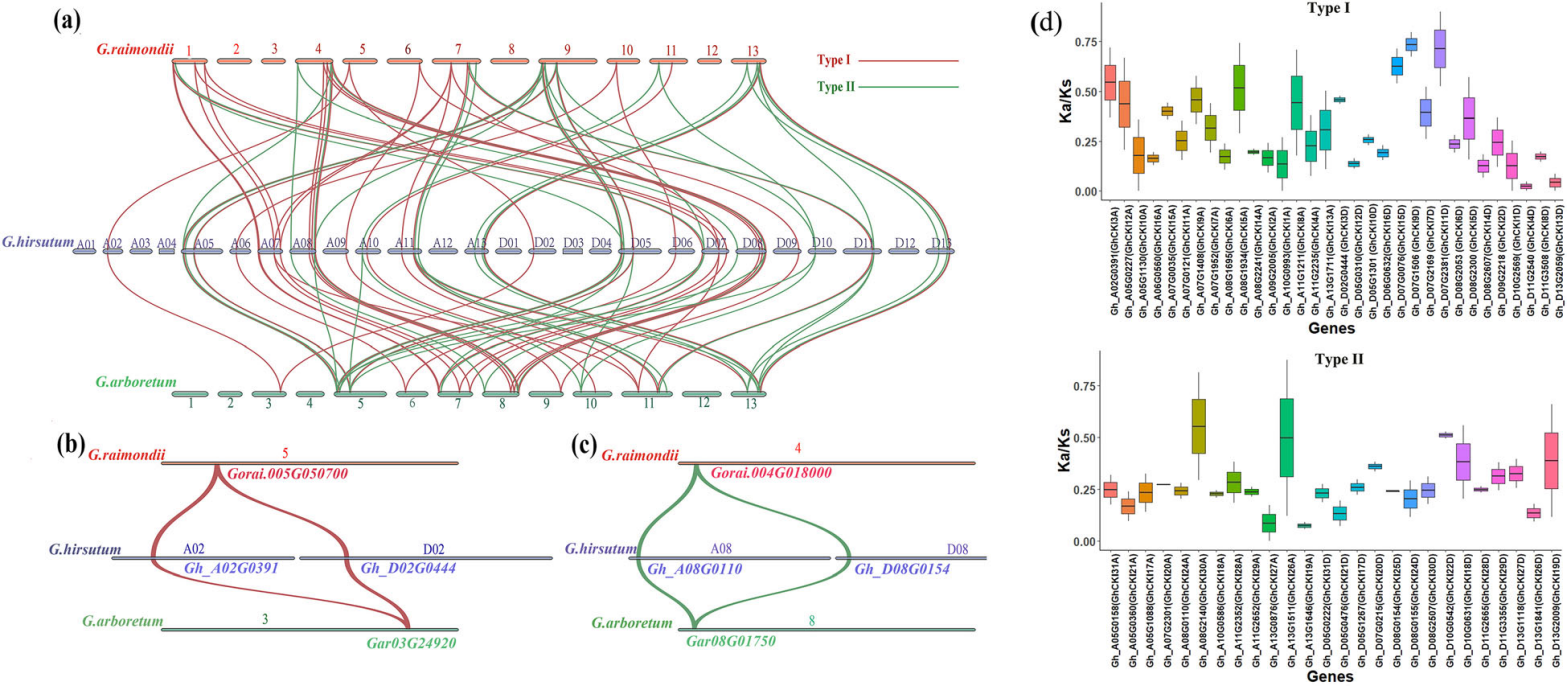
Collinear analysis among *G. hirsutum, G. arboreum, G. raimondii* and the distribution of Ka/Ks values of two types *CKI* genes. **a** Collinear analysis of chromosome fragments containing 20 adjacent genes upstream and downstream of two types of *CKI* genes among *G. hirsutum*, *G. arboreum*, *G. raimondii.***b**-**c** A case of collinearity for a type I paralogous gene pairs and type II paralogous gene pairs respectively. Red for type I, green for type II. **d** The Ka/Ks values of homologous gene pair between diploid (*G. arboretum* and *G. raimondii)* and tetraploid (*G. hirsutum)* of two types *CKI* genes. *G. hirsutum* genes as reference. The above was the result of type I *CKI*, the below was the result of type II *CKI*

Four duplication events have been detected in cotton from the ancient angiosperms development period to the stage of tetraploid cotton species: an ancient whole genome duplication (WGD) very early in angiosperm evolution, one triplication event, the recent WGD event specific for cotton, and the tetraploid event [25, 27, 28]. Based the phylogenetic tree we constructed (Fig. 1a), *GhCK*I might expanded by hybridization of diploid A genome species and D genome species [29]. To further examine the evolutionary history of the *GhCKI* genes, we firstly carried out gene collinearity analysis between diploid genome (A genome: *G.arboreum* and D genome: *G. raimondii)* and tetraploid genome (AD genome: *G.hirsutum*) (Fig. 3a). The collinearity of chromosomal regions which contained *CKI* genes indicated that these genes were the products of WGD or segment duplication. Clearly, both most of genes of two types have a homologous gene in diploid genome and two homologous in tetraploid genome. For example, type I gene *GrCKI3* (*Gorai.005G050700*) on D genome and *GaCKI* (*Gar03G24920*) on A genome expanded from one copy to two copies *GhCKI3A* (*Gh_A02G0391*) and *GhCKI3D* (*Gh_D02G0444*) on the AD genome (Fig. 3b). Type II gene *GrCKI25* (*Gorai.004G018000*) on D genome and *GaCKI* (*Gar08G01750*) on A genome expanded from one copy to two copies *GhCKI25A* (*Gh_A08G0110*) and *GhCKI25D* (*Gh_D08G0154*) on the AD genome (Fig. 3c). These results revealed that both type I and type II *CKI* genes were replicated in cotton tetraploid event. In addition, we investigated the selection pressure of two types *CKI* genes by calculating the ratio of nonsynoymous substitutions per nonsynonymous site (Ka) to synonymous substitutions per synonymous site (Ks) (omega) for each homologous gene pair in three above *Gossypium* species (Table S2). Genes in *G. hirsutum* as reference. We found all *CKI* genes were under negative selection (Fig. 3d). The result showed that all *CKI* genes were highly conserved during the evolution of *Gossypium*, which show the importance of *CKI* genes for *Gossypium*.

### The origin and divergence speculation of two types *CKI* family

**Fig. 4:**
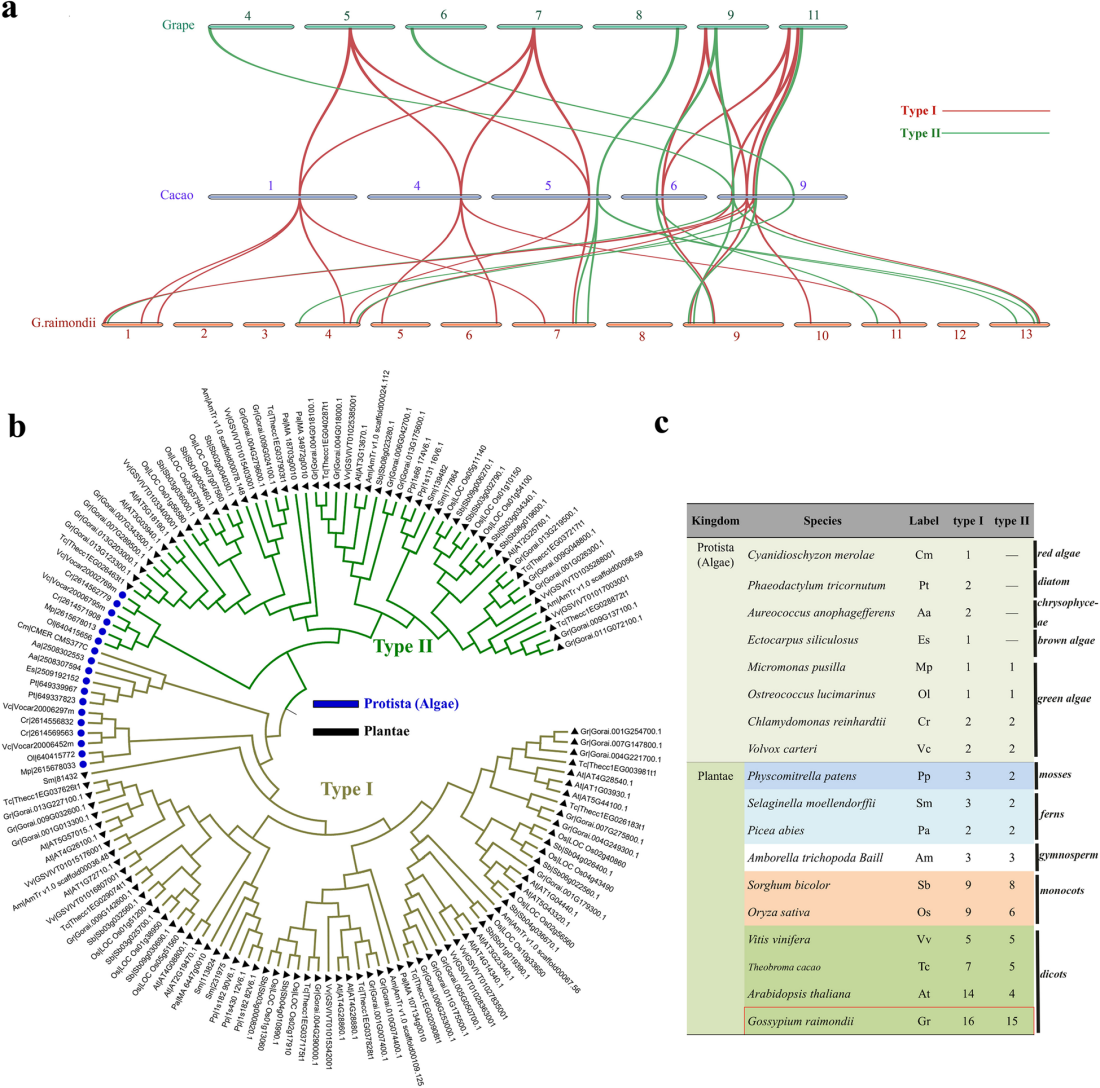
Collinear analysis among grape, cacao, *G. raimondii* and phylogenetic tree and number of the Casein Kinase I (*CKI*) in eighteen species. **a** Collinear analysis of chromosome fragments containing 20 adjacent genes upstream and downstream of two types *CKI* genes among grape, cacao, *G.raimondii.***b** Phylogenetic tree of the Casein Kinase I (*CKI*) in eighteen species. Blue dot represent algae of Protista, Black triangle represent plants of Plantae. **c** The number of two types *CKI* genes in eighteen species

Cotton was diverged from an ancestor shared with *Theobroma cacao* at least 60 Myr ago, and individual grape chromosome segments resembling ancestral eudicot genome structure [27]. To further explore the evolutionary history of the two types *CKI* genes, we first studied the evolution between three dicotyledons: grape, cacao, cotton (Fig. 4a). As observed in cotton, there were two types of *CKI* syntenic relationships among the collinearity of grapes, cocoa, and cotton. Therefore, the divergence of two types *CKI* genes should be earlier than the formation of dicots. Interestingly, it can be observed that there were more type I *CKIs* than type II *CKIs* among the collinearity of grape to cacao (Fig. 4a). Maybe this was why there were more type I *CKI* genes than type II *CKI* genes in cotton. Then phylogenetic analysis between the *G. raimondii* and *Arabidopsis* (eudicots) and rice (monocot) were carried out (Figure S4). Clearly, two types *CKI* genes from eudicots (*G. raimondii* and *Arabidopsis*) and monocot (rice) were present in all subgroups (Figure S4). These results indicating that the divergence of two type *CKI* genes in plants precedes the divergence between monocots/eudicots.

To better trace the origin of the two types *CKI* genes divergence, *CKI* genes from 18 species, including algae, mosses, ferns, gymnosperms, monocotyledons and dicotyledons, were found to construct phylogenetic trees (Table S1, Table S3). It can be clearly detected that *CKI* genes of all species were also divided into two types (Fig. 4b). And the extremely absorbing results were found when we counted the number of two type *CKI* genes respectively (Fig. 4c). Type I *CKI* genes were present in all species, but type II genes were lost in algae besides green algae. According to some reports, red algae and green algae diverged about 1,500 MYA ago [30], then other algae were produced by secondary endosymbiosis [31], so the result above may imply that type I genes arose at a very ancient time and type II *CKI* genes were newly generated upon the divergence of red alga and green algae. Besides, two types *CKI* homologs in plant including mosses, ferns, gymnosperms, monocot and dicot showed a tendency to increase from lower to higher plants, the result means that after the divergence of red algae and green algae, green algae produced the type II *CKI* genes in the subsequent evolutionary process, then two type of *CKI* genes expanded in the subsequent evolutionary process to form the two type of *CKI* family in various plant today.

### Analysis of cis-acting elements of *GhCKI* and *GhCKI* expression profile in different tissues from *G. hirsutum*

Cis-acting elements play an important role in gene expression regulation in plant. We found many cis-acting elements in the promoters of *GhCKI* genes and identified four main types of cis-acting elements: light responsive element, abiotic stress responsive element, plant hormones responsive element, plant growth and development related element (Figure S5). This result indicates that CKI were involved in many biological processes through different cis-acting element. To further determine which tissue CKI mainly plays a role in cotton, the expression profiles in different organs/tissues (including roots, stems, leaves, petals, anthers, and 5 DPA [days post anthesis] ovules) were examined by quantitative RT-PCR (qRT-PCR) in *G. hirsutum*. Because of the high sequence similarity between A subgenome and D subgenome cDNAs and regulatory region of homologous genes, so we named the 58 putative *G. hirsutum CKI* genes as *GhCKI1A/D* to *GhCKI31A/D.* We designed one common primer pair for analyzing *CKIA/D* gene expression. After the specificity for each primer pair was verified, suitable qRT-PCR primer pairs for 44 (26 type I *CKI* genes and 18 type II *CKI* genes) of the 61 *CKI* genes (33 type I *CKI* genes and 28 type II *CKI* genes) were obtained (Table S4). As shown in Figure S6, the most of the type I and type II *CKI* genes exhibited different tissue expression. *GhCKI2A/D*, *GhCKI3A/D*, *GhCKI8A/D*, *GhCKI10A/D*, *GhCKI11A/D*, *GhCKI14A/D*, *GhCKI15A/D*, *GhCKI18A/D*, *GhCKI20A/D*, and *GhCKI27A/D* were constitutively expressed in every tested tissue, implying that these genes may play regulatory roles at multiple developmental stages. In addition, some genes were highly relatively expressed in leaf and anther, such as *GhCKI4A/D*, *GhCKI14A/D*, *GhCKI19A/D*, *GhCKI20A/D*, *GhCKI27A/D*, *GhCKI28A/D*, *GhCKI29A/D*, *GhCKI30A/D*. Some of these genes, such as *GhCKI4A/D*, *GhCKI14A/D*, *GhCKI20A/D*, and *GhCKI27A/D,* were preferentially expressed in leaves. Interestingly, these genes were also relatively conserved in evolution and may have earlier differentiation times (Fig. 3d, Table S2). These results provide additional insight into their roles during different growth and development processes in cotton.

### Circadian rhythm and light signal regulation of *GhCKI* genes expression

**Fig. 5:**
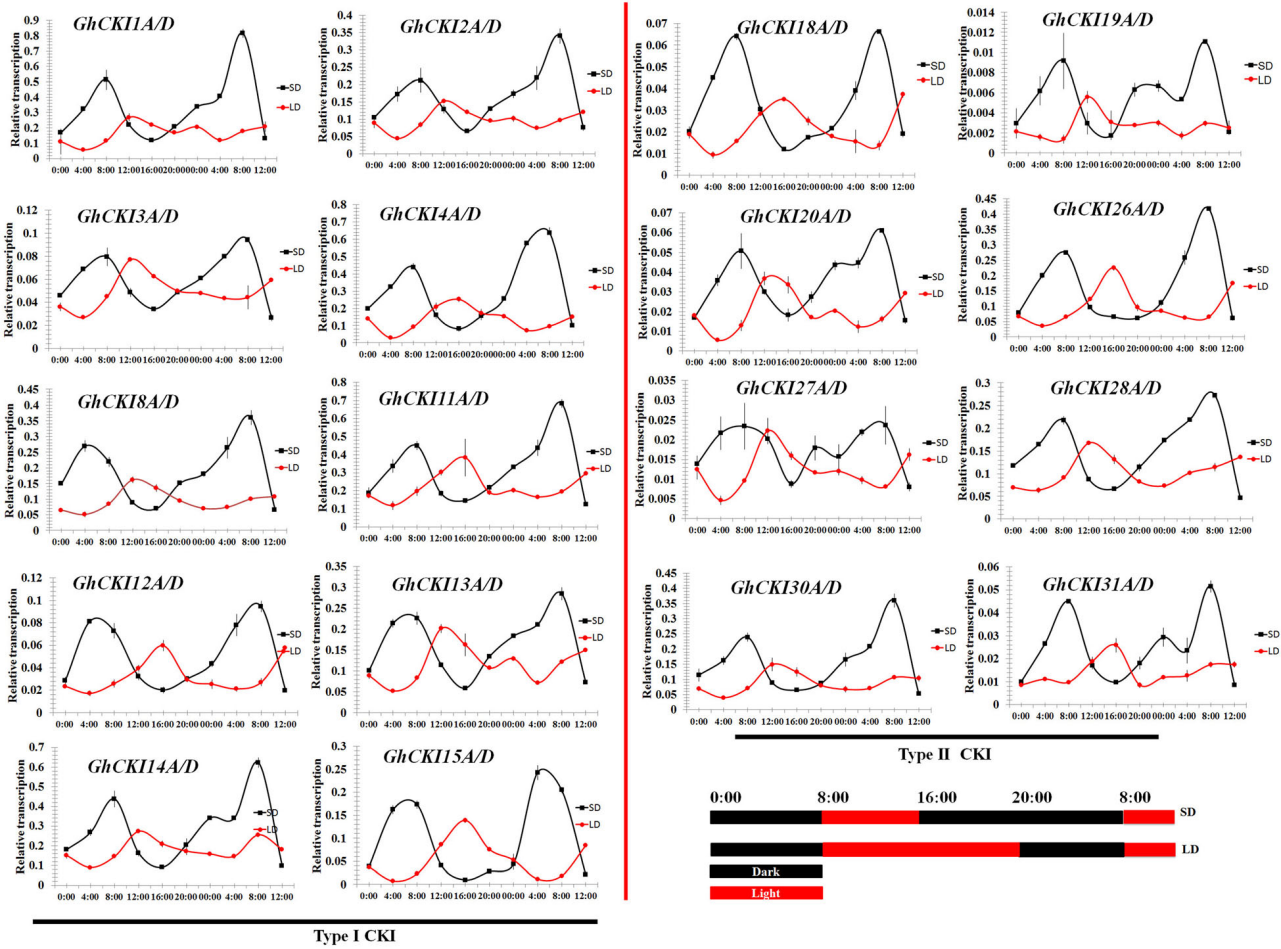
Expression profiles of two types *GhCKI* genes under different diurnal conditions. **a** and **b** Expression profiles of the type I and type II *CKI* genes in *G. hirsutum* under different diurnal conditions, respectively. Diurnal changes in transcript levels of 36 *G. hirsutum CKI* genes under short-day (SD) and long-day (LD) conditions. SD, short day (8 h light); LD, long day (16 h light); Black boxes denote dark, while the solid red boxes show light. Values are means ± standard deviations. Asterisks indicate statistically significant differences (*P < 0.05, **P < 0.01) by Student’s *t*-test. The *GhUB7* (*Gh_A11G096*) was used as the reference gene to normalize the total amount of cDNA in each reaction

After the divergence of green algae and red algae [30], green algae further evolved into higher plants that adapt to terrestrial life [32]. In this process, adaptation to biological processes such as light and circadian rhythm occured. Besides, analysis of cis-acting elements shows both types of *GhCKI* have many light-responsive components and plant growth and development related element including circadian rhythm (Figure S5). So *CKI* expression studies on circadian rhythms and light responses were performed. To determine whether the expression of cotton two types *CKI* genes is regulated by photoperiod (circadian clock), the transcription level of *G. hirsutum CKI* genes under different diurnal conditions were investigated. Thirty-six *G. hirsutum CKI* genes were expressed at a sufficient level to evaluate their circadian regulation (Fig. 5). Under short-day (SD) conditions, all the 36 *CKI* genes showed a high expression peak at 8:00, and the expression of *GhCKI* genes increased gradually in the dark and decreased in the light (Fig. 5). Under long-day (LD) conditions, twelve type I genes (*GhCKI1A/D*, *GhCKI2A/D*, *GhCKI3A/D*, *GhCKI8A/D*, *GhCKI13A/D*, and *GhCKI14A/D*) and ten type II genes (*GhCKI19A/D*, *GhCKI20A/D*, *GhCKI27A/D*, *GhCKI28A/D*, and *GhCKI30A/D*) showed a high expression peak at 12:00. However, the expression peaks of eight type I genes (*GhCKI4A/D*, *GhCKI11A/D*, *GhCKI12A/D*, and *GhCKI15A/D*) and six type II genes (*GhCKI18A/D*, *GhCKI26A/D*, and *GhCKI31A/D*) were at 16:00 under long-day (LD) conditions (Fig. 5), and *GhCKI14D* and *GhCKI26D* also were relatively conserved in evolution (Fig. 3d, Table S2). In addition, the expression of the 36 *GhCKI* genes was higher in the light than in the dark under LD conditions, was different with these genes under SD, LD seems to simulate an increase in the duration of sunlight during evolution from red algae to green algae. These results identified a set of clock-regulated *GhCKI* genes showing different phases of expression and provided additional insight to understand the underlying mechanisms of modulation in circadian rhythm.

**Fig. 6:**
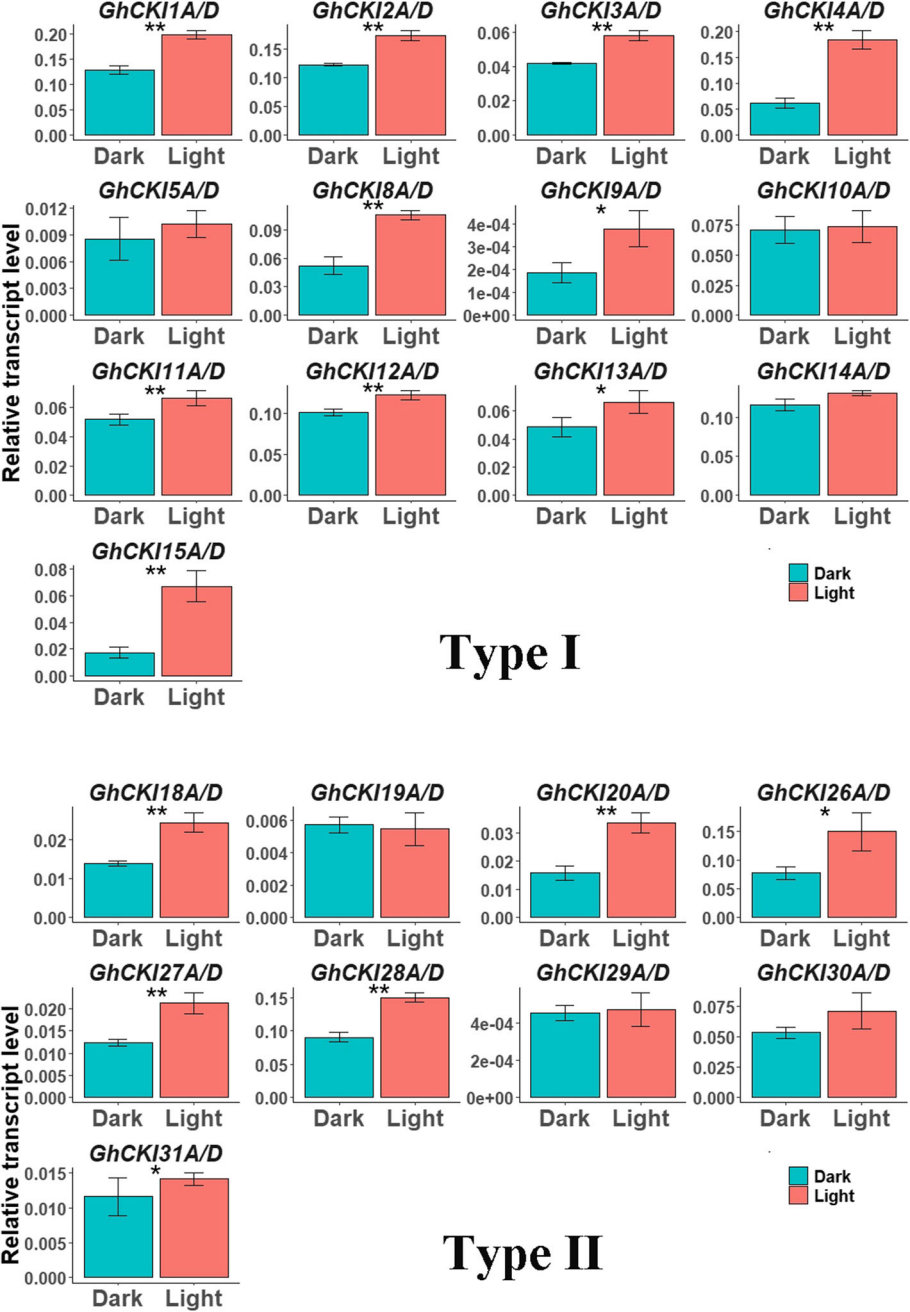
Expression profiles of two types *GhCKI* genes under dark and light conditions. **a** and **b** Expression profiles of the type I *GhCKI* genes (26 genes) and type II *GhCKI* genes (18 genes) in upland cotton cotyledons under dark and light conditions, respectively. Error bars indicate ± standard deviations of triplicate experiments. Asterisks indicate statistically significant differences (*P < 0.05, **P < 0.01) by Student’s *t*-test. The *GhUB7* (*Gh_A11G096*) gene was used as the reference gene to normalize the total amount of cDNA in each reaction

To further investigate whether two types *CKIs* are involved in light signaling, the expression of *G. hirsutum* (Fig. 6) *CKI* genes and *G. raimondii* (Figure S7, Table S5) in cotyledon under light and dark conditions was examined by qRT-PCR. The expression of most *CKI* genes both in *G. hirsutum* and *G. raimondii* was up-regulated under light, except for *GhCKI19A/D* (Fig. 6), *GrCKI6*, *GrCKI14*, *GrCKI25*, and *GrCKI26* (Figure S7), there was only a slight difference between the two types of *CKI* genes. The results indicated both type I and type II *CKI* might involve in plant light signal. However, intriguingly, genes expressed preferentially in leaves respond more strongly to light signals, such as *GhCKI4A/D*, *GhCKI20A/D*, *GhCKI27A/D*, implying the evolutionarily conserved *CKI* genes may play a more important role in the response of cotton to light signals.

### *CKI* genes respond to high temperature during cotton anther development process

**Fig. 7:**
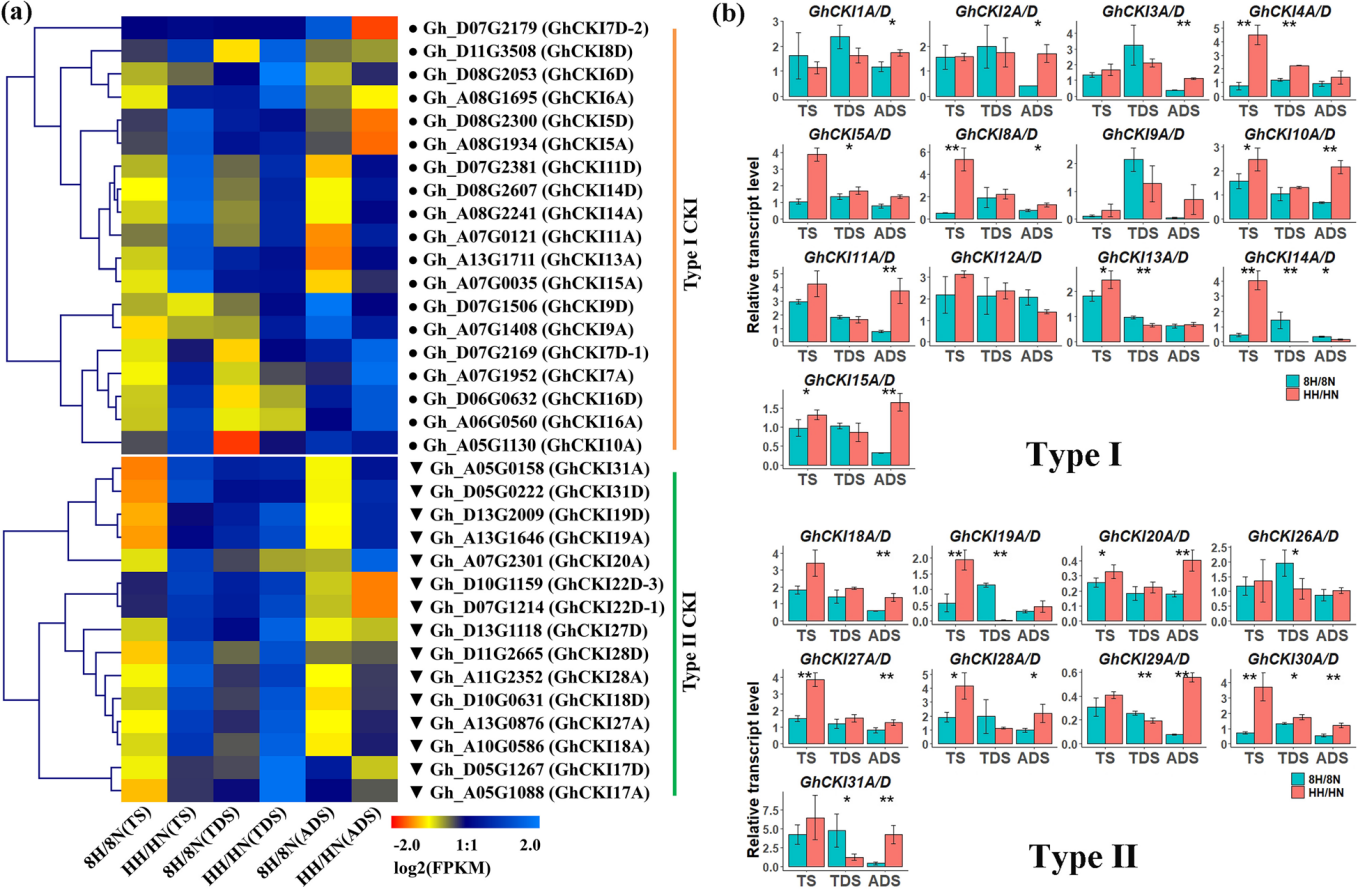
Expression profiles of two types *G. hirsutum CKI* genes in anthers at different development stages under NT and HT conditions. **a**-**b** Expression patterns of *G. hirsutum CKI* genes in 84,021 and H05 at different anther developmental stages under NT and HT conditions from RNAseq (**a**) and Quantitative RT-PCR experiment (**b**). 8 N and 8H refer to 84,021 (the HT-tolerant line) under NT and HT conditions, respectively; HN and HH refer to H05 (the HT-sensitive line) under NT and HT conditions, respectively; TS: tetrads stage; TDS: tapetal degradation stage; ADS, anther dehiscence stage; FPKM: fragments per kb of transcript per million fragments mapped. The RNAseq data of the expression profiles was cited from Min et al. [33]. Error bars indicate ± standard deviations of triplicate experiments. Asterisks indicate statistically significant differences (*P < 0.05, **P < 0.01) by Student’s t-test. The *GhUB7* (*Gh_A11G096*) was used as the reference gene to normalize the total amount of cDNA in each reaction

*CKI* genes exhibited the highest expression in cotton anthers, except for *GhCKI14A/D* and *GhCKI27A/D* (Figure S6)*. CKI* promoter also contain numerous abiotic stress responsive cis-acting element (Figure S5). Meanwhile, our previous studies showed that one member of the *CKI* gene family, *GhCKI* (*Gh_A07G0121*/*GhCKI11A*) was induced in H05 (the high temperature (HT)-sensitive line) anthers, but not in 84,021 (the HT-tolerant line) anthers under HT condition [19]. Genome-wide analyses of *G. hirsutum CKI* genes in response to HT during anthers development may lay a foundation for further understanding the mechanisms involved in HT tolerance or HT sensitivity. In the present study, a heat-map representing expression profiles was produced using the transcriptomic data which contained the three different anther development stages (TS, tetrad stage; TDS, tapetal degradation stage; ADS, anther dehiscence stage, the three most sensitive periods to high temperature) of 84,021 and H05 under HT and NT [33]. It was observed that 34 of 61 *GhCKI* genes (19 from type I and 15 from type II) were differently expressed in 84,021 and H05 under HT and NT, even after filtering with an absolute threshold log_2_ (fold-change) ≥ 1 (Fig. 7a). We found that most genes were up-regulated in H05 after HT exposure, such as type I genes (*GhCKI10* and *GhCKI11*) and type II genes (*GhCKI20* and *GhCKI27*) (Fig. 7a). To verify the result, the qRT-PCR experiments were also performed (Fig. 7b), consistently with the RNA-seq data, most CKI genes were up-regulated in H05 under HT condition. We also analyzed the HT induced differentially alternative spliced (AS) of *CKI* genes based on our RNA-seq data [33]. Several CKI genes have HT induced differentially AS event (Figure S8, Table S6), for instance, *Gh_A10G0586* (*GhCKI18A*) have a retained intron event, *Gh_A11G1211* (*GhCKI8A*) have a skipped exon event (Figure S8, Table S6). These results indicated that HT influenced the expression and alternative splice of *GhCKI* genes during anther development.

## Discussion

### Structural characteristics of *G. hirsutum CKI* genes

In animals, certain characteristics of *CKI* that certainly influence its activity have been identified at the protein level: structure-related regulation, subcellular localization, interaction with other proteins, and post-translational modifications [34]. As a member of the superfamily of serine/threonine-specific kinases, the function of phosphorylation is a priority to focus on. In plant, some research found *CKI* family divided into two types, type I was canonical casein kinase 1, type II was plant-specific casein kinase 1 [12, 13]. However, systematically identification and characteristics of cotton *CKI* genes were not analyzed. In this study, we first systematically identified *CKI* genes in five sequenced cotton species (*G. raimondii, G. arboreum, and G. hirsutum, G.barbadense, G.herbaceum*) (Fig. 1a). Based on sequence comparison and phylogenetic analysis, cotton *CKI* genes were divided into two types similar to other reports, namely type I (canonical casein kinase 1) and type II (plant-specific casein kinase 1) *CKI* genes respectively (Fig. 1a). Motif compositions, and exon/intron distribution patterns in terms of exon number in *G. hirsutum* agreed with our hypothesis (Fig. 2).

The Ser/Thr kinase domain contains putative kinase catalytic loop, substrate recognition region and ATP-binding site. Regarding the functional characteristics of the N-terminal and conserved C-terminal regions, previous reports in mammals showed that CKI presents a β-sheets N-terminal lobe and mainly a α-helical C-terminal lobe, which are connected by a hinge region forming a catalytic cleft for substrate and ATP binding [35, 36]. Within the C-terminal region, a specific phosphate moiety binding motif has been identified allowing the recognition of phosphorylated protein substrates, which is believed to be involved in *CKI* regulatory interactions. These reports showed that the N-terminal and C-terminal lobes play an important role in substrate phosphorylation. In this study, type I CKI proteins resulted highly conserved within their kinase domains, but significantly differing in the length and primary structure of their C-terminal domains (Figure S1, Figure S3), which is consistent with previous reports [3, 4, 25, 33, 37]. Interestingly, contrary to type I, type II CKI proteins also presented conserved kinase domains and possessed variable N-terminal and conserved C-terminal regions (Figure S1, Figure S3). Based on the characteristic of plant *CKI* genes, we hypothesis the type I and type II *CKI* genes possess different functions on account of the difference in structures, especially regarding the function of phosphorylation. Functional studies will be needed in the future to further explore this difference.

### Evolutionary expansion of the two types *CKI* gene family

The evolution of *Gossypium* was characterized by a history of multiple gene duplications at different stages as in the four duplication events (an ancient angiosperm WGD event, one triplication event, a specific and recent cotton WGD event, and the tetraploid event) [25, 28, 29]. First, we observed the expansion of the two types *CKI* genes in cotton (Fig. 3a), and found that the two types *CKI* genes were expanded with cotton tetraploid event. Most genes were one copy in diploid and became two copies in tetraploid. But the collinearity of three cotton species chromosomal regions were not contained all *CKI* genes. For example, *Gh_D10G2569* (type I) and *Gh_D10G0542* (type II) didn’t have homologous gene in synteny blocks between *G. raimondii* and *G. hirsutum or G. arboretum* and *G. hirsutum* (Fig. 3a). We speculated these genes were newly created copies during the cotton tetraploid event, or errors in genome assembly. In order to better trace the origin and divergence of the two types *CKI* genes, the genome of 18 species were collected to construct the phylogenetic tree. We found that *CKI* genes in all species were divided into two types, and red algae did not have type II gene (Fig. 4). But type II *CKI* genes were identified in green algae (Fig. 4), consistent with previous studies [38]. After red algae and green algae diverged about 1,500 MYA ago [30], other algae were produced by secondary endosymbiosis [31]. Then the green alga evolved into plant [32]. Therefore, we speculate that the two type *CKI* genes diverged about 1,500 MYA ago, then *CKI* genes have replicated and expanded differently in different species during the evolution of green algae to higher plants. Besides, the gene families which possess an ancient evolutionary history have important functions, such as *MADS-box* genes [39], *Auxin response factor*s [40], or *ASYMMETRIC LEAVES2-LIKE/ LOB-DOMAIN* transcription factors [41]. Thus, we speculate that *CKI* genes may play important roles in plant development and be involved in very diverse biological roles to adapted to environmental stress.

### Expression patterns of the *GhCKI* genes

To date, although the functions of only one *CKI* gene have been characterized in tetraploid cotton [19], no systematic functional analysis of expression patterns for different groups of the tetraploid cotton *CKI* gene family was done. In this study, we demonstrated that *CKI* genes displayed expression divergence in different tissues (Figure S6). For instance, *GhCKI2A/D*, *GhCKI3A/D*, *GhCKI8A/D*, *GhCKI10A/D*, *GhCKI11A/D*, *GhCKI15A/D,* and *GhCKI18A/D* were constitutively expressed in every tested tissue (such as roots), implying that these genes may play regulatory roles at multiple development stages. In *Arabidopsis*, *AtCKL2* and *AtCKL3* were required for ABA regulation of seed germination, root growth, and gene expression [16, 17]. In rice, *OsCKI1* deficiency resulted in shorter primary roots and fewer lateral and adventitious roots [2]. Similar expression patterns suggest that these preferentially or specifically expressed *G.hirsutum CKI* genes might play important roles in root formation and development. Among these 61 identified *CKI* genes, *GhCKI* (namely *GhCKI11A*) is the only one that was speculated to regulate tapetal programmed cell death and anther dehiscence in cotton [19]. Under HT condition, *AtCKL2* and *AtCKL7* were expressed in the tapetum, in anther microspores at stages 9–12, and in anther pollen grains at stages 13–14, which imply *AtCKL2* and *AtCKL7* may be key regulators of tapetal development under HT [42]. Apart from *GhCKI11A* (formerly *GhCKI*), the *GhCKI1A/D*, *GhCKI5A/D*, *GhCKI9A/D*, *GhCKI12A/D*, *GhCKI13A/D*, *GhCKI19A/D*, *GhCKI26A/D*, *GhCKI28A/D*, *GhCKI29A/D*, *GhCKI30A/D*, and *GhCKI31A/D* genes were exclusively expressed in anther. This finding suggested that *CKI* genes were components of a complex transcriptional network regulating anther development.

Previous studies had showed that HT stress causes premature programmed cell of the tapetum, resulting in male sterility and catastrophic loss of crop production [30, 43–45]. However, the mechanism underlying successful male reproductive development under HT remains largely unknown. Except for the *GhCKI11A* (formerly *GhCKI*) gene, which was reported to regulate tapetum development under HT [20], no other *CKI* genes have demonstrated to participate in the regulation of anther development. In this study, the expression of type I and type II *CKI* genes were analyzed in cotton anther respond to HT, no differences between type I and type II *CKI* genes were found (Fig. 7). Thus, two types *CKI* genes both participated in regulating stamen development under HT and that will be employed in future works.

Circadian clocks are molecular timekeepers. Many plants use photoperiod (circadian clock) information to prepare for daily environmental changes and increase their fitness in changing environments [46]. The circadian clocks share similar network architecture of feedback loops that form by transcriptional and post-translational regulation among the clock components [47, 48]. Phosphorylation is a common post-translational modification and is an integral part of circadian regulation [49]. Another function of *CKI* is to take a series of biological process via phosphorylating various substrates [3]. In *Arabidopsis*, both *CK1.3* and *CK1.4* showed a high expression peak before dark under LD conditions, which indicated that the expression of *CK1.3* and *CK1.4* was strictly regulated by a circadian rhythm; overexpression of either *CK1.3* or *CK1.4* delayed flowering under LD conditions [13]. However, in cotton, it is not clear that the systematic function of the tetraploid cotton *CKI* gene family is involved in photoperiod (circadian clock). In our present study, the expression of *CKI* genes is circadian. Under long-day (LD) conditions, the expression in the light is higher than in the dark. Under short-day (SD) conditions, the expression of all the 36 *CKI* genes showed a high expression in dark. (Fig. 5). The results indicated that the expression of *CKI* was strictly regulated by a circadian rhythm. According to these evidences, we propose that at least some *CKI* genes are components of a diurnal rhythm complex network. The functions of these *GhCKI* genes in regulating the circadian clock on the diurnal rhythms will be further characterized in future works.

Plant reception of light signal and its subsequent reaction is important for both growth and development. In *Arabidopsis*, *CK1* genes are involved in the light signaling pathway mainly through phosphorylation; casein kinase 1 proteins CK1.3 and CK1.4 phosphorylate CRYPTOCHROME 2 (CRY2) which is the blue-light receptor, to regulate blue light signaling [13]. Our results showed that the expression of most type I and type II *CKI* genes was upregulated under light conditions compared to dark (Fig. 6, Figure S7). We thus believe that both type I and type II *CKI* gene are involved in the response to light signals. But we do not know whether they participate in light signals by phosphorylating photoreceptors CRY2 or light signal components, including HY5, HF5, HFR1, COP1, and PIF1, which in *Arabidopsis* are phosphorylated by casein kinase 2 proteins [49]. We also do not know whether the pathways and capabilities of these two types of genes are the same.

We found cis-acting element of *GhCKI* involve light responsive element, abiotic stress responsive element, plant hormones responsive element, plant growth and development related element, so we carried two types of cotton *CKI* expression analysis about high temperature response, biological rhythm, and light response. The results of expression analysis indicated that *GhCKI* genes may involve in the above biological functions. Therefore, *GhCKI* maybe participate in these biological functions through different cis-elements. Although the expression patterns of type I and type II *CKI* genes were basically the same in our study, we believe that these two types of genes are functionally differentiated because of the diversity in structure, which requires further investigations in the future.

## Conclusion

Our study offers a promising landscape to unravel the underlying structural characteristics and evolutionary expansion of cotton *CKI* genes and further elucidate their expression patterns in different tissues and various conditions; this is crucial to better understand their characteristics and to elucidate their precise functions in regulating various facets of plant growth and development.

## Materials and methods

### Database search and identification of *CKI* genes

The genome information of twenty-two species was retrieved from the website shown in Table S3. The *CKI* genes in *A.thaliana* were downloaded from TAIR (www.arabidopsis.org) database. The Hidden Markov Model (HMM) was built with 17 *Arabidopsis* CKI protein by using hmmbuild command of HMMER (v3.2.1) [50]. The protein databases of the remaining twenty-one species were searched with hmmsearch command (-E 1e-20) [50]. All possible *CKI* genes were identified in these twenty-one species. To further ensure the accuracy of each *CKI* gene, we identified the conserved kinase domains (domain id is: http://pfam.xfam.org/family/PF00069) present in possible CKI proteins using SMART (http://smart.embl-heidelberg.de/), Pfam (http://pfam.xfam.org). Only gene containing the conserved kinase domains considered as true *CKI*.

### Chromosomal location, gene structure and phylogenetic analysis

The chromosomal location of *GhCKIs* were obtained from cotton genome database (https://www.cottongen.org/), then gene duplication events of *GhCKIs* were detected based on principle described in previous study [51], moreover the Circos-0.69 Software was used to visualize chromosomal location and gene duplication [52]. Sequence alignments were generated with CLUSTALX [53], and the alignments among CKIs were adjusted before the tree was constructed. The online Gene Structure Display Server 2.0 [54] (http://gsds.cbi.pku.edu.cn/) was used to identify the exon/intron organization. *G.hirsutum* CKI protein sequences were submitted to online MEME (Multiple EM for Motif Elicitation) program [55] (http://meme-suite.org/tools/meme) to identify conserved protein motifs. Phylogenetic trees were constructed by the maximum likelihood (ML) method, the Poisson correction model, complete deletion and bootstrap analysis performed with 2,000 replicates [56].

### Collinearity analysis and the estimation of selection pressure of *CKI* genes

The chromosome location of *CKI* genes in each genome was obtained according to the GFF3 file. When homologous *CKI* genes were located in the same chromosome with no more than one intervening gene, these genes were defined as tandem duplication genes. We used the method as described in Maher et al. to identify large-scale duplication events [57]. If two genes were located in the same duplication block, the protein sequences on their flanks were highly similar at the amino acid level. Therefore, we located the *CKI* genes on the genome and used this location as the initial anchor site to obtain 20 protein coding genes upstream and downstream of each site respectively [56]. This chromosomal region containing 41 protein-coding genes were selected for collinearity analysis using the python version of MCScan [58].

The coding sequences were aligned and guided by alignments of protein sequences using the PAL2NAL software with the NOGAPS parameter [59]. The yn00 procedure in PAML package was used to calculate the ratio of nonsynonymous substitutions per nonsynonymous site (Ka) to synonymous substitutions per synonymous site (Ks) for each homologous gene pair [60]. According to the definition of Ka/Ks, values less than one represent negative or purifying selection, while values greater than one represent positive selection. The saturation effect was ruled out by removing gene pairs with Ks > 2.5.

### Analysis of cis-acting elements in *GhCKI* promoter

The promoter sequences of the *GhCKI* genes (1,000 bp upstream of the initiation codon “ATG” were extracted from the cotton geome sequences (https://cottonfgd.org/), PlantCARE (http://bioinformatics.psb.ugent.be/webtools/plantcare/html/search_CARE.html) was used to predict the cis-acting elements of the *GhCKI* promoter sequence [61], the light responsive element, abiotic stress responsive element, plant hormones responsive element, plant growth and development related element were analyzed.

### High temperature-induced differential alternative spliced *CKI* gene detection

All putative high temperature-induced differential AS event were extacted from our RNA-seq data using rMATS [33, 62]. After weakly expressed AS events were filtered below 40% samples with total IJC + SJC ≥ 30, differentially AS events between NT and HT condition were identified using rMATS if the difference in the PSI of AS event between two conditions exceeds a stringent threshold (FDR ≤ 0.05, ΔPSI ≥ 10%). Those *CKI* genes containing AS event considered as high temperature-induced differential AS *CKI* Gene.

### Plant materials, growth conditions and stress treatments

Four cotton accessions used in this experiment are provided by Huazhong Agricultural University. For the expression profiles in different organs/tissues, various samples of *G. hirsutum* cv. YZ1 were extracted from roots, stems, leaves, petal, anther, and ovules excised carefully from bolls five DPA. To analysis the expression patterns of *Gossypium hirsutum CKI* genes at different anther developmental stages under NT and HT conditions, two cotton (*Gossypium hirsutum*) lines with obvious differences in performance under HT were employed in this study: 84,021, which is tolerant to HT, and H05, which is sensitive to HT [33]. The plants were grown in a greenhouse at 28 °C to 35 °C/20 °C to 28 °C day/night as a normal condition. During HT treatment, the plants were cultivated at 35 °C to 39 °C/29 °C to 31 °C day/night in a greenhouse. When the plants were treated with HT for 7 d, buds of different lengths (6–7, 9–14, and more than 24 mm) were collected under HT and NT. The anthers were excised and immediately frozen in liquid nitrogen; they were then stored at -80 °C until use. The transcriptome profiles of *CKI* genes were isolated from the RNA-seq data [33]. In order to analyze the diurnal regulation of *G. hirsutum CKIs* gene expression, *G. hirsutum* cv. YZ1 was grown under short-day conditions (8 h light/16 h dark) and long-day conditions (16 h light/8 h dark), respectively. The leaves of four leaves period cotton plants were harvested. We also collected cotyledons under light and dark conditions, that from *G. raimondii* and *G. hirsutum* cv.YZ1 respectively.

### qRT-PCR

Various plant samples were collected and immediately frozen in liquid nitrogen and stored at -80 °C. total RNA was isolated from the collected cotton tissues using previously published methods [63]. First-strand cDNA was generated from 3 μg total RNA using the M-MLV reverse transcriptase (Invitrogen). The cDNA was used as a template for qRT-PCR. The qRT-PCR reactions were performed using the 7500 Real-Time PCR System (Applied Biosystems). The primers used in this study were listed in Table S4, Table S5.

## Supplementary Information

**Additional file 1: Supplementary Figure S1 to S5.** **Figure S1.** Multiple sequence alignments of two type GhCKI proteins. **Figure S2.** Structural analysis of the *CKI* genes in *G. hirsutum*. **Figure S3.** Multiple sequence alignments of two types GrCKI proteins. **Figure S4.** Phylogenetic analyses of *CKI* gene family in eudicot (*A. thaliana, G. raimondii*) and monocot (*O. sativa*). **Figure S5.** Cis-acting elements on the promoter of *CKI* genes in *G. hirsutum*. **Figure S6.** Quantitative RT-PCR analysis of the expression of *G. hirsutum CKI* genes in upland cotton tissues. **Figure S7.** Expression profiles of *CKI* genes under dark and light condition in *G. raimondii*. **Figure S8.** Two examples of high temperature induced differentially alternative spliced *CKI* genes.

**Additional file 2: Table S1.** The gene ID of *CKI* genes in 22 species.

**Additional file 3: Table S2.** The value of nonsynoymous substitutions per nonsynonymous site (Ka) and synonymous substitutions per synonymous site (Ks) for *CKI* homologous gene pair in cotton.

**Additional file 4: Table S3.** Summary of 22 sequenced genomes used for identification of *CKI* genes.

**Additional file 5: Table S4.** Primer for Quantitative RT-PCR analysis of *GhCKI* genes expression in *G. hirsutum.*

**Additional file 6: Table S5.** Primer for Quantitative RT-PCR analysis of *GrCKI* genes expression in *G.raimondii.*

**Additional file 7: Table S6.** High temperature induced differentially spliced *CKI* genes

## Data Availability

The data that support the results are included within the article and its additional files. Other relevant materials are available from the corresponding authors on reasonable request.

## Abbreviations

CKI: Casein kinase I
ABA: Abscisic acid
EL1: Early flowering 1
Hd16: Heading data 16
PCD: Programmed cell death
MLK3: Mut9-like kinases 3
CKL: Casein Kinase 1-like
Gr: *G. raimondii*
Gh: *G. hirsutum*
WGD: Whole genome duplication
DPA: Days post anthesis
HT: High temperature
NT: Normal temperature
TS: Tetrad stage
TDS: Tapetal degradation stage
ADS: Anther dehiscence stage
LD: Long-day
SD: Short-day
