# Supplementary material for: Genome-wide identification, evolutionary estimation and functional characterization of two cotton CKI gene types
Source: BMC Plant Biol. 2021 May 22;21:229. doi: 10.1186/s12870-021-02990-y (PMC8140429; doi:10.1186/s12870-021-02990-y)
Supplement: Supplementary file 1 — Additional file 1: Supplementary Figure S1 to S5. Figure S1. Multiple sequence alignments of two type GhCKI proteins. Figure S2. Structural analysis of the CKI genes in G. hirsutum. Figure S3. Multiple sequence alignments of two types GrCKI proteins. Figure S4. Phylogenetic analyses of CKI gene family in eudicot (A. thaliana, G. raimondii) and monocot (O. sativa). Figure S5. Cis-acting elements on the promoter of CKI genes in G. hirsutum. Figure S6. Quantitative RT-PCR analysis of the expression of G. hirsutum CKI genes in upland cotton tissues. Figure S7. Expression profiles of CKI genes under dark and light condition in G. raimondii. Figure S8. Two examples of high temperature induced differentially alternative spliced CKI genes. [file 12870_2021_2990_MOESM1_ESM.pdf]

Genome-wide identification, evolutionary estimation and functional characterization of two cotton *CKI* gene types

Yanlong Li[1, a], Yaoyao Li[1, 2, a], Yuanyuan Chen[1], Maojun Wang[1], Jing Yang[3], Xianlong Zhang[1], Longfu Zhu[1], Jie Kong[3*], Ling Min[1*]

[1]National Key Laboratory of Crop Genetic Improvement, Huazhong Agricultural University, Wuhan 430070, Hubei, China

[2]College of Life Sciences, State Key Laboratory for Conservation and Utilization of Subtropical Agro-Bioresources, South China Agricultural University, Guangzhou 510642, Guangdong, China

[3]Institute of Economic Crops, Xinjiang Academy of Agricultural Sciences, Xinjiang 830091, China

[a]These authors contributed equally

[*]Corresponding Authors: lingmin@mail.hzau.edu.cn and kongjie.258@163.com

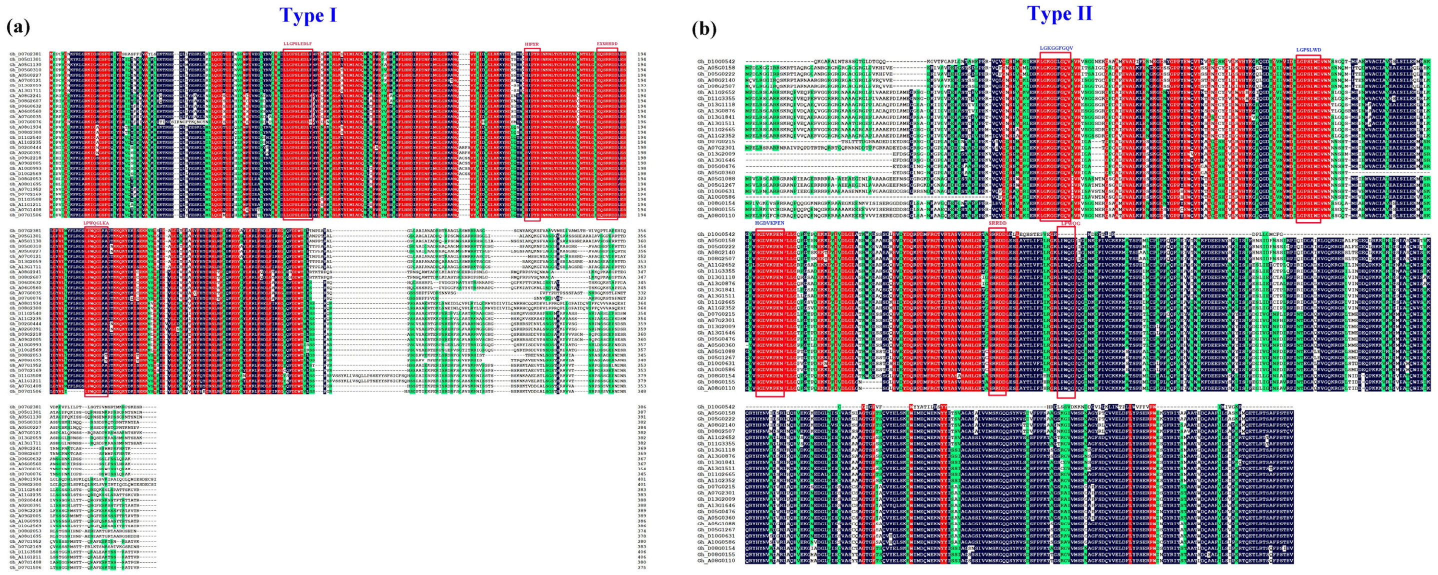

Figure S1. Multiple sequence alignments of type I CKI proteins (a) and type II CKI proteins (b)

(a) **Type I**

Gene ID	N-terminal(aa)	Kinase domain(aa)	C-terminal(aa)
Gh_D09G2218	1-8	9-232	233-389
Gh_A09G2005	1-8	9-232	233-389
Gh_A10G0993	1-8	9-232	233-386
Gh_D10G2569	1-8	9-232	233-386
Gh_D02G0444	1-8	9-246	247-388
Gh_A02G0391	1-8	9-246	247-388
Gh_A08G1934	1-8	9-243	244-402
Gh_D08G2300	1-8	9-243	244-402
Gh_D11G2540	1-8	9-243	244-383
Gh_A11G2235	1-8	9-243	244-383
Gh_D08G2053	1-8	9-240	241-374
Gh_A08G1695	1-8	9-240	241-378
Gh_A07G1952	1-8	9-230	231-380
Gh_D07G2169	1-8	9-230	231-383
Gh_D11G3508	1-8	9-240	241-406
Gh_A11G1211	1-8	9-240	241-406
Gh_A07G1408	1-8	9-240	241-380
Gh_D07G1506	1-8	9-154	155-375
Gh_D05G1301	1-8	9-230	231-387
Gh_A05G1130	1-8	9-230	231-391
Gh_D05G0310	1-8	9-230	231-382
Gh_A05G0227	1-8	9-230	231-384
Gh_D13G2059	1-8	9-229	230-382
Gh_A13G1711	1-8	9-229	230-382
Gh_A07G0121	1-8	9-230	231-382
Gh_D07G2381	1-8	9-230	231-386
Gh_A08G2241	1-8	9-257	258-369
Gh_D08G2607	1-8	9-257	258-369
Gh_D06G0632	1-8	9-257	258-367
Gh_A06G0560	1-8	9-234	235-367
Gh_A07G0035	1-8	9-234	235-354
Gh_D07G0076	1-8	9-259	260-345

(b) **Type II**

Gene ID	N-terminal(aa)	Kinase domain(aa)	C-terminal(aa)
Gh_A05G0158	1-69	70-316	317-549
Gh_A05G0360	1-35	36-255	256-487
Gh_A05G1088	1-78	79-326	327-558
Gh_A07G2301	1-78	79-322	323-555
Gh_A08G0110	1-78	79-319	320-551
Gh_A08G2140	1-69	70-316	317-549
Gh_A10G0586	1-8	9-255	256-488
Gh_A11G2352	1-78	79-325	326-558
Gh_A11G2652	1-78	79-325	326-558
Gh_A13G0876	1-78	79-326	327-559
Gh_A13G1511	1-78	79-325	326-558
Gh_A13G1646	1-36	37-279	280-512
Gh_D05G0222	1-69	70-316	317-549
Gh_D05G0476	1-35	36-279	280-511
Gh_D05G1267	1-78	79-326	327-558
Gh_D07G0215	1-50	51-262	263-527
Gh_D08G0154	1-78	79-325	326-557
Gh_D08G0155	1-78	79-319	320-551
Gh_D08G2507	1-69	70-316	317-549
Gh_D10G0542	1-47	48-206	207-331
Gh_D10G0631	1-79	80-326	327-559
Gh_D11G2665	1-78	79-353	354-558
Gh_D11G3355	1-78	79-325	326-558
Gh_D13G1118	1-78	79-325	326-558
Gh_D13G1841	1-78	79-325	326-558
Gh_D13G2009	1-36	37-279	280-512

Figure S2. Structural analysis of the type I *CKI* genes (a) and type II *CKI* genes (b) in *G. hirsutum*.

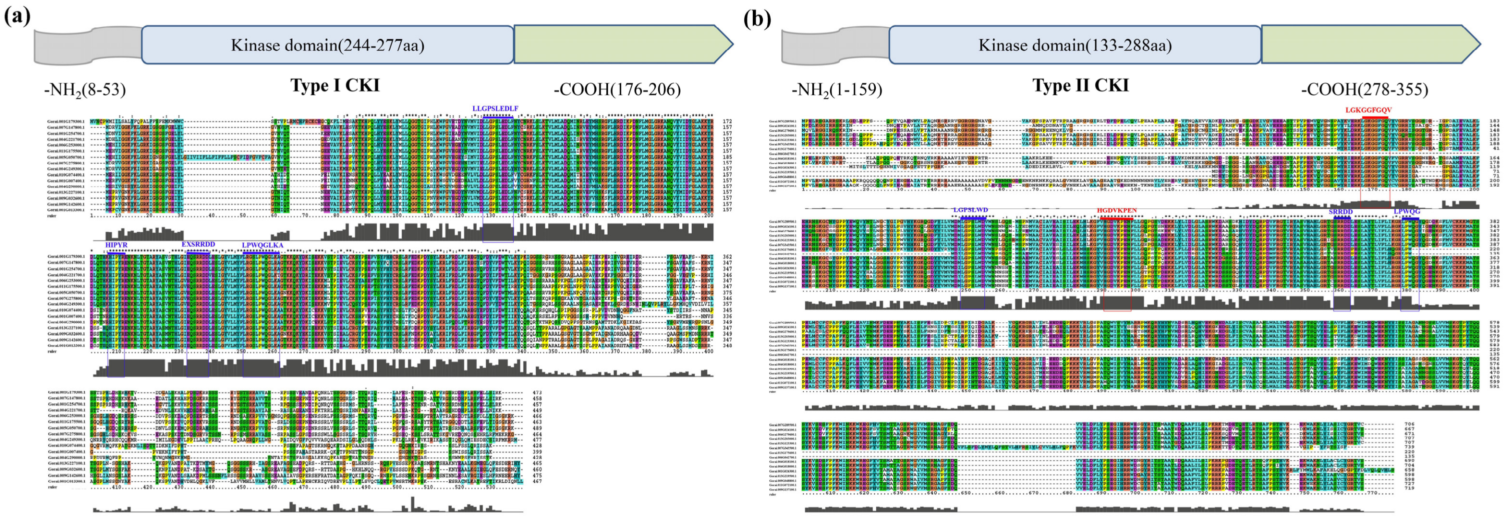

Figure S3. Multiple sequence alignments of type I GrCKI proteins (a) and type II GrCKI proteins (b).

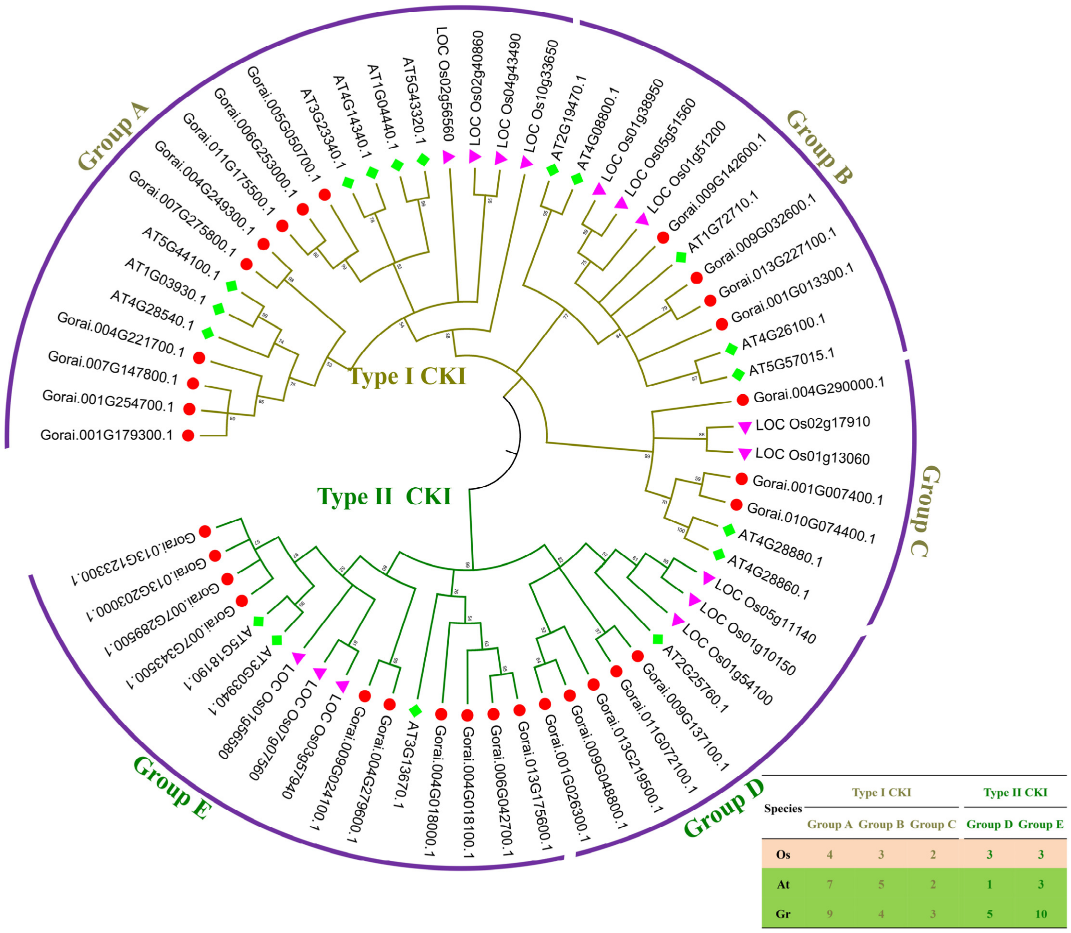

Species	Type I CKI			Type II CKI	
	Group A	Group B	Group C	Group D	Group E
Os	4	3	2	3	3
At	7	5	2	1	3
Gr	9	4	3	5	10

Figure S4. Phylogenetic analyses of *CKI* gene family in eudicot (*A. thaliana, G. raimondii*) and monocot (*O. sativa*) (Table S1). Amino acid sequences were aligned using ClustalX and the phylogenetic tree was conducted using MEGA 6 software with the maximum likelihood method. Three different color were represented for the three species, red for *G. raimondii*, green for *A. thaliana*, and purple for *O. sativa*.

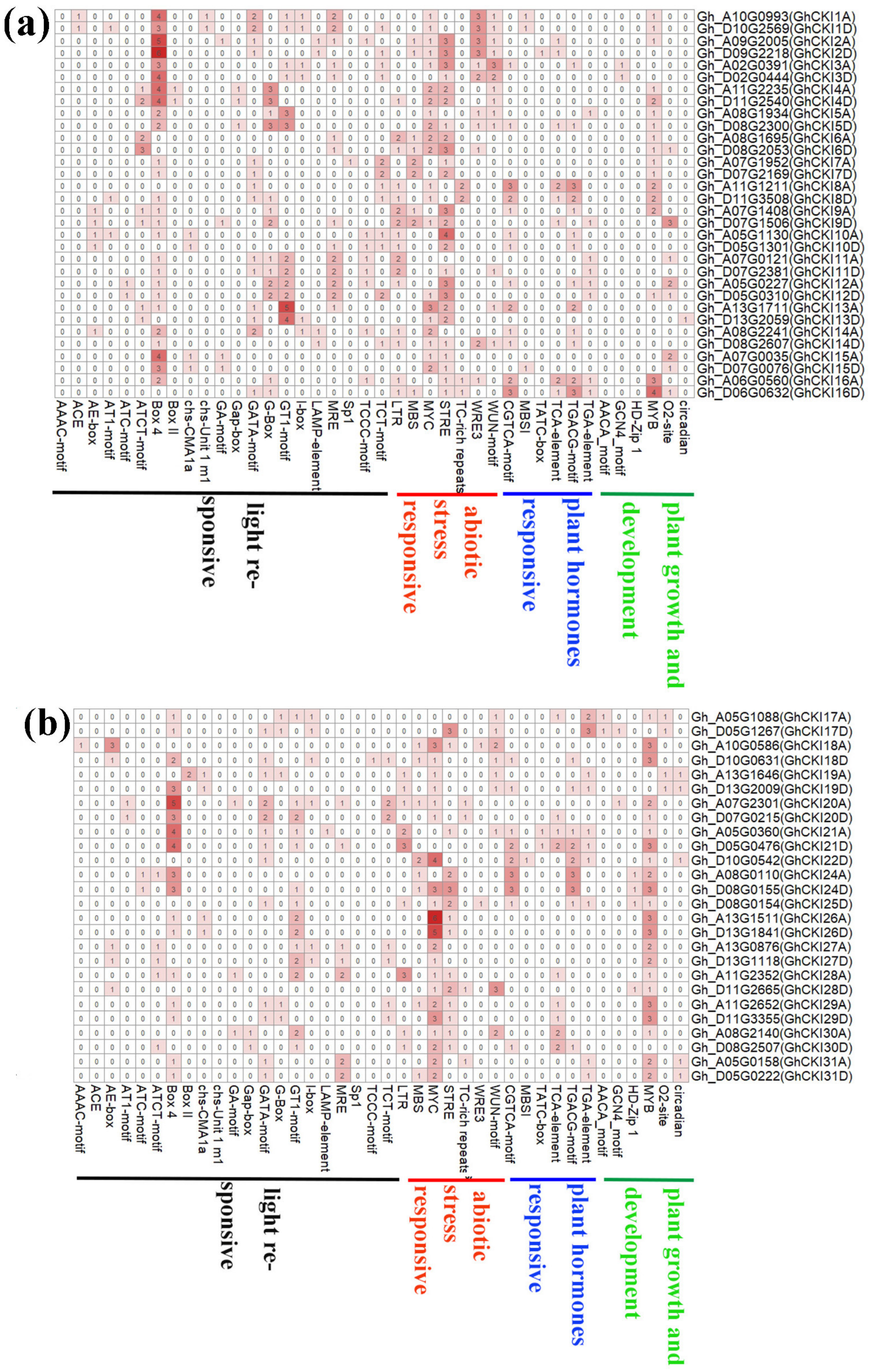

Figure S5. Cis-acting elements on the promoter of type I *CKI* genes (a) and type II *CKI* genes (b) in *G. hirsutum*. Black for light responsive element, red for abiotic stress responsive element, blue for plant hormones responsive element, green for plant growth and development related element.

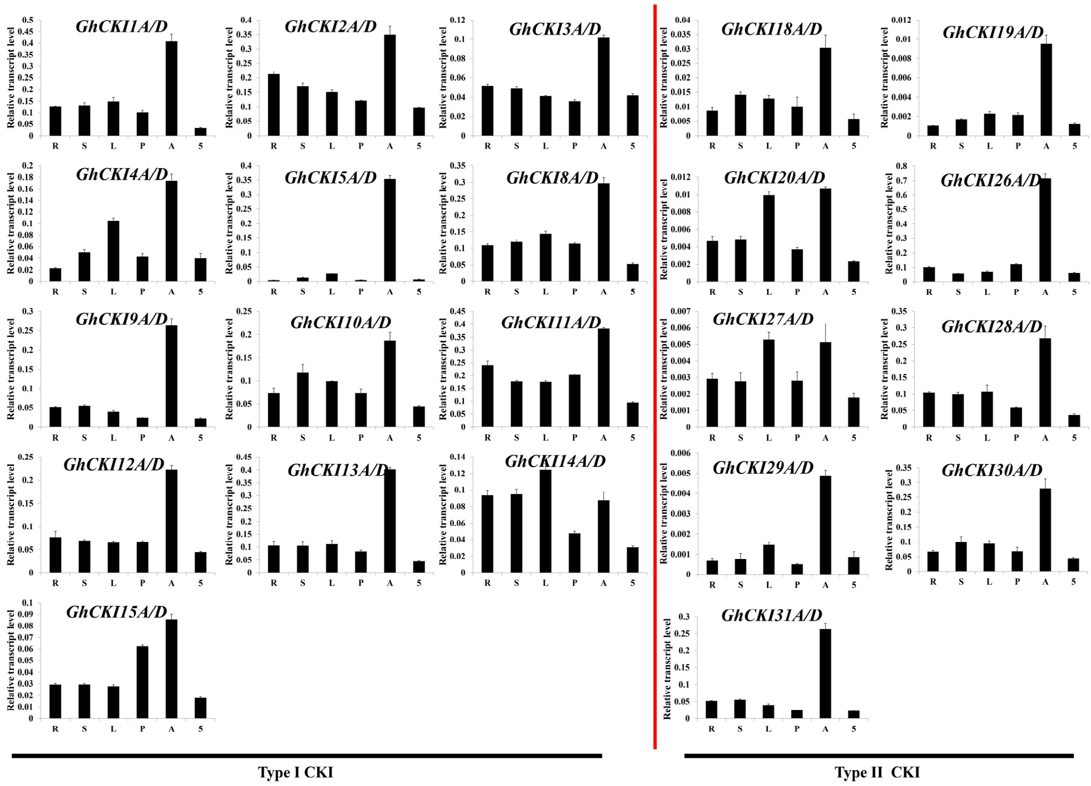

Figure S6. Quantitative RT-PCR analysis of the expression of *G. hirsutum CKI* genes in upland cotton tissues. Expression profiles of *GhCKI* genes in upland cotton tissues. The left is the type I *GhCKI* genes (26 genes), the right is the type II *GhCKI* genes (18 genes). R, root; S, shoot; L, leaves; P, petal; A, anther; and 5, ovules at 5 days post-anthesis (DPA). The *GhUB7* (*Gh_A11G096*) gene was used as the reference gene to normalize the total amount of cDNA in each reaction.

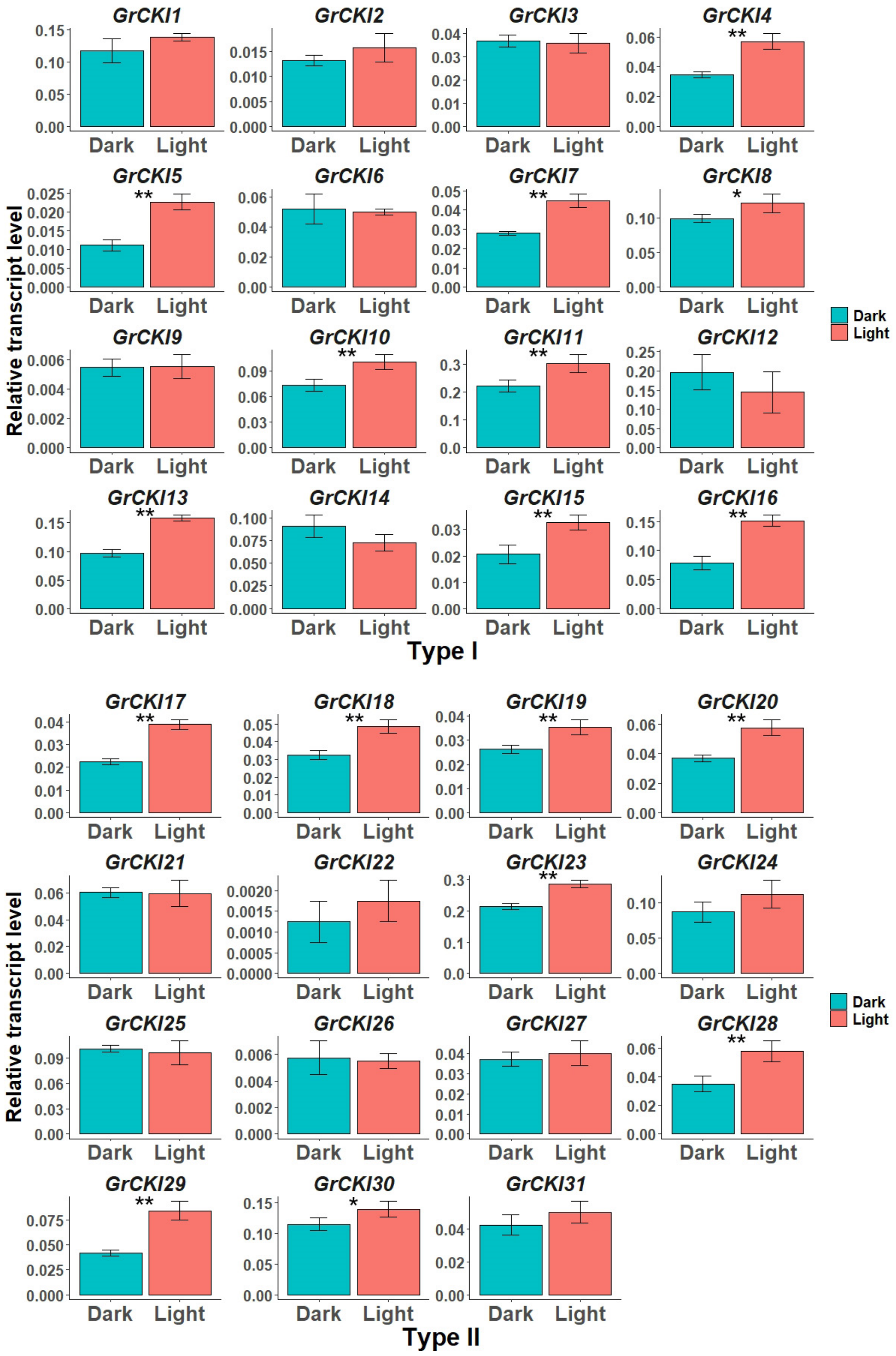

Figure S7. Expression profiles of *CKI* genes under dark and light condition in *G. raimondii*. Error bars indicate ± standard deviations of triplicate experiments. Asterisks indicate statistically significant differences (*P<0.05, **P<0.01) by Student's t-test. The *GrUB7* (*Gorai.011G04910*) gene was used as the reference gene to normalize the total amount of cDNA in each reaction.

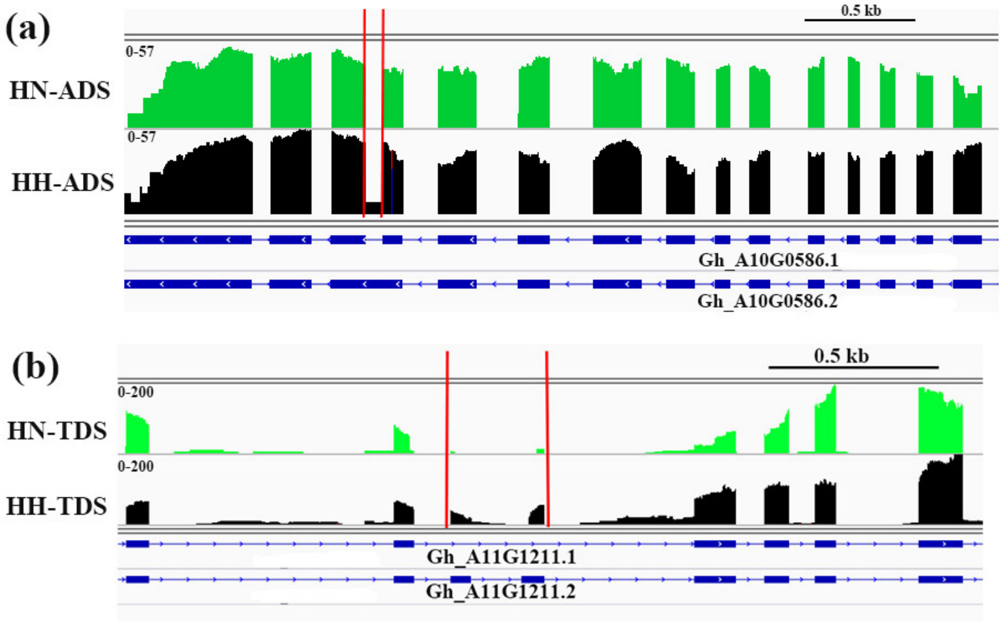

Figure S8. Two examples of high temperature induced differentially alternative spliced CKI genes. (a) A retained intron (RI) event between HN-ADS and HH-ADS of *Gh_A10G0586* (*GhCKI18A*). (b) A skipped exon (SE) event between HN-TDS and HH-TDS of *Gh_A11G1211* (*GhCKI8A*). HN and HH refer to H05 (the HT-sensitive line) under NT and HT condition, respectively. TDS: tapetum degradation stage; ADS, anther dehiscence stage; Representation of AS differences between NT and HT detected by RNA-seq using the Integrative Genomics Viewer (https://software.broadinstitute.org/software/igv/).
